# Comprehensive Analysis of VEGFR2 Expression in HPV-Positive and -Negative OPSCC Reveals Differing VEGFR2 Expression Patterns

**DOI:** 10.3390/cancers13205221

**Published:** 2021-10-18

**Authors:** Senem Uzun, Yüksel Korkmaz, Nora Wuerdemann, Christoph Arolt, Behrus Puladi, Oliver G. Siefer, Hanife G. Dönmez, Martin Hufbauer, Baki Akgül, Jens P. Klussmann, Christian U. Huebbers

**Affiliations:** 1 Jean-Uhrmacher-Institute for Otorhinolaryngological Research, University of Cologne, 50937 Cologne, Germany; Senem.Uzun@uni-duesseldorf.de (S.U.); oliver.siefer@uk-koeln.de (O.G.S.); 2 Department of Periodontology and Operative and Preventive Dentistry, University Medical Center of the Johannes Gutenberg University, 55131 Mainz, Germany; koyuekse@uni-mainz.de; 3 Department of Otorhinolaryngology, Head and Neck Surgery, University Hospital of Cologne, 50937 Cologne, Germany; nora.wuerdemann@uk-koeln.de; 4 Centre for Molecular Medicine Cologne (CMMC), Faculty of Medicine, University of Cologne and University Hospital Cologne, 50931 Cologne, Germany; 5 Institute for Pathology, University Hospital of Cologne, 50937 Cologne, Germany; christoph.arolt@uk-koeln.de; 6 Department of Oral and Maxillofacial Surgery, University Hospital RWTH Aachen, 52074 Aachen, Germany; bpuladi@ukaachen.de; 7 Institute of Virology, University of Cologne, Medical Faculty and University Hospital Cologne, 50935 Cologne, Germany; hanifetanir@hacettepe.edu.tr (H.G.D.); martin.hufbauer@uk-koeln.de (M.H.); baki.akguel@uk-koeln.de (B.A.); 8 Department of Biology, Hacettepe University, Ankara 06800, Turkey

**Keywords:** vascular endothelial growth factor receptor 2, oropharyngeal squamous cell carcinoma, human papillomavirus, cancer stem cell

## Abstract

**Simple Summary:**

Up to 50% of oropharyngeal squamous cell carcinomas (OPSCC) are associated with human papillomavirus type 16 (HPV16), the annual incidence of which is steadily increasing. HPV-positive and -negative OPSCC exhibit a different biology, which is characterized by distinct mutation signatures and expression patterns. It is known that VEGFR2 is commonly overexpressed in HNSCC, but the influence of HPV on VEGFR2 in OPSCC is still unknown, although VEGFR2 has emerged as a promising target in tumor therapy. The aim of our study was to evaluate whether HPV exerts specific effects on VEGFR2 expression in OPSCC and thus possibly on the regulation of vascularization. Interestingly, while HPV-negative carcinoma upregulates VEGFR2 in tumor cells, in HPV-positive carcinoma VEGFR2 is upregulated in tumor-supporting blood vessels. HPV-positive OPSCC with high VEGFR2 expression is associated with poor prognosis, supporting the prognostic significance of deregulated VEGF signaling for OPSCC patients.

**Abstract:**

VEGF signaling regulated by the vascular endothelial growth factor receptor 2 (VEGFR2) plays a decisive role in tumor angiogenesis, initiation and progression in several tumors including HNSCC. However, the impact of HPV-status on the expression of VEGFR2 in OPSCC has not yet been investigated, although HPV oncoproteins E6 and E7 induce VEGF-expression. In a series of 56 OPSCC with known HPV-status, VEGFR2 expression patterns were analyzed both in blood vessels from tumor-free and tumor-containing regions and within tumor cells by immunohistochemistry using densitometry. Differences in subcellular colocalization of VEGFR2 with endothelial, tumor and stem cell markers were determined by double-immunofluorescence imaging. Immunohistochemical results were correlated with clinicopathological data. HPV-infection induces significant downregulation of VEGFR2 in cancer cells compared to HPV-negative tumor cells (*p* = 0.012). However, with respect to blood vessel supply, the intensity of VEGFR2 staining differed only in HPV-positive OPSCC and was upregulated in the blood vessels of tumor-containing regions (*p* < 0.0001). These results may suggest different routes of VEGFR2 signaling depending on the HPV-status of the OPSCC. While in HPV-positive OPSCC, VEGFR2 might be associated with increased angiogenesis, in HPV-negative tumors, an autocrine loop might regulate tumor cell survival and invasion.

## 1. Introduction

A rising proportion of head and neck squamous cell carcinomas (HNSCC) localized in the oropharynx (oropharyngeal squamous cell carcinoma, OPSCC) is associated with human papillomavirus (HPV) infections with HPV16 being the most prevalent type [1]. Data from the United States allowed for the projection that OPSCC case numbers would overtake the number of cervical carcinomas in 2020 and data from the Centers for Disease Control (CDC) show that this was already the case in 2019 [1,2].

Based on their differing risk factors, clinicopathological presentation, biological profiles, mutation patterns and expression signatures, HPV-positive and HPV-negative OPSCC can be regarded as two distinct entities [3]. Accordingly, the recent TNM-Classification of OPSCC has been adapted and now distinguishes between p16^INK4A^-positive (HPV-driven) and p16^INK4A^-negative (HPV-negative) OPSCC, thus taking different treatment prognoses into account [4].

The vascular endothelial growth factor receptors (VEGFRs) are expressed on the cell surface and bind to the signaling ligand vascular endothelial growth factor (VEGF). VEGFR2 on endothelial cells is primarily involved in angiogenesis and plays a crucial role in tumor angiogenesis [5]. However, VEGFR2 is not only expressed on endothelial cells but can also be observed in tumor cells [6]. Apart from VEGFR2, two other VEGF receptors are described to be of clinical significance. VEGFR1 is localized on immune and endothelial cells and is considered as a decoy receptor that limits the amount of free available VEGF ligand. VEGFR3 is explicitly expressed in the lymph endothelium and is therefore crucial for lymph vessel formation [7]. Following binding of the VEGF ligand, the VEGF/VEGFR2 signal cascade is induced and leads to activation of pathways such as PLC-ERK1/2, PI3K-AKT-mTOR, of Src tyrosine kinases, small GTPases and kinases such as MAPK and STATs [5]. The activated signaling pathways regulate endothelial cell migration, cell proliferation and cell motility, and lead to an increase in nitric oxide (NO) production, thus regulating vascular tone and permeability [8]. VEGFR2-signalling is responsible for the formation, function and maintenance of vessels, all physiological processes which decisively contribute to the nutrient supply in healthy tissue as well as tumors [5]. Therefore, VEGFR2 is an important control node for tumor growth. Strategies to interact with the vascular supply in a therapeutic approach have been studied over the past decades, and VEGFR2 has emerged as a promising target in tumor therapy [8].

HNSCC, in general, present with overexpression of VEGFR2 and have the potential to create an autocrine loop, which is characterized by the tumor cells’ ability to control their proliferation, motility, invasive capacity and survival in response to VEGFR2 expression [9,10]. However, the impact of HPV status on the quantitative and qualitative expression of VEGFR2 has not yet been investigated, although HPV may have specific effects on VEGFR2 expression. In vitro studies with HPV-positive and -negative cervical cancer cell lines have shown that the viral oncoproteins E6 and E7 induce VEGF-expression [11,12]. Although E6 degrades the transcription factor TP53, which acts as an angiogenic suppressor, E6 is capable of inducing VEGF in a TP53 independent manner by direct interaction with its promoter region [11]. Furthermore, both E6 and E7 promote Hypoxia-inducible factor 1 alpha (HIF-1α) expression, which can upregulate VEGF, while knockdown of HIF-1α has been shown to suppress angiogenic activity in viral oncogene expressing cells in vitro [12].

Furthermore, VEGF expression is induced by the transcription factor nuclear factor erythroid-derived 2-like 2 (NFE2L2 / NRF2) [13]. Upon oxidative stress (OS) stimuli, NRF2 dissociates from its cytosolic inhibitor complex consisting of Kelch-like ECH-associated protein 1(Keap1), Cullin-3 (Cul3) and Ring-Box 1 (RBX1) to translocate into the nucleus, where NRF2 binds to antioxidant response element-like (ARE) sequences in promoter regions of several target genes including VEGF. The role of NRF2 is to protect normal cells from damage induced by reactive oxygen species (ROS) and to contribute to tissue regeneration [14]. However, hyperactivation of NRF2 followed by overexpression of several of its target genes leads to exuberant protection against OS in malignant cells preventing apoptosis and cell death, eventually leading to resistance against radio- and chemotherapy [13,15]. In HPV-negative HNSCC, NRF2 overexpression is typically a consequence of mutations and copy number variations in its own gene and in the genes Keap1, Cul3 and RBX1 encoding for its regulatory complex, whereas NRF2 deregulation is caused by viral proteins in HPV-positive tumors [15,16,17]. Aldo-Keto-Reductase 1C3 (AKR1C3) is one of these target genes upregulated by NRF2 that, in addition to its role in lipid metabolism, is a phase I detoxifying enzyme for numerous drugs, including platinum-type chemotherapeutic agents [14]. AKR1C3 expression is a useful read-out for detecting increased OS levels in various cancers including OPSCC, whereby overexpression correlates with significantly unfavorable survival [14,15].

## 2. Materials and Methods

### 2.1. Patient Material and Ethics Statement

**Figure 1:**
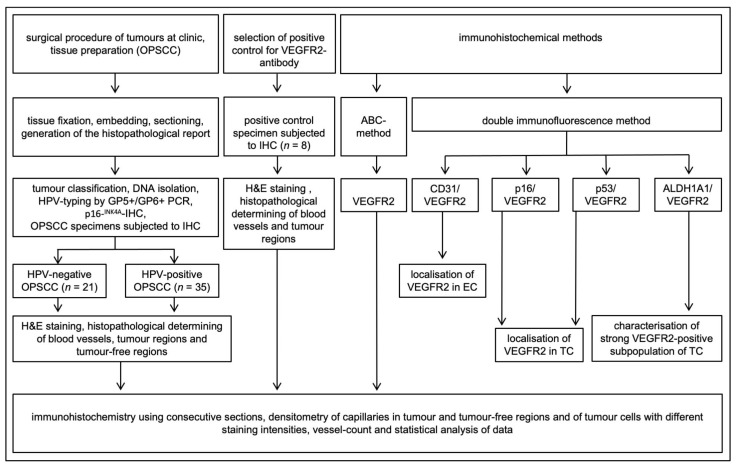
Schematic flow diagram of the study design and method. After surgery and tissue processing of OPSCC samples, the HPV-status was determined by GP5+/GP6+ polymerase chain reaction (PCR) and p16INK4A-immunohistochemistry (IHC). To test the specificity of the VEGFR2-antibodies, liver, melanoma, papillary thyroid carcinoma, and cervix squamous cell carcinoma tissues were selected as positive controls. First, hematoxylin and eosin (H&E) staining was performed to determine the regions relevant for further analyses. This was followed by staining of consecutive sections with VEGFR2-AB using the avidin-biotin-peroxidase complex (ABC). Subsequently, densitometric analyses of the VEGFR2-staining intensity of blood vessels in tumor regions and tumor-free regions, as well as tumor cells, were performed. The number of VEGFR2-expressing capillaries in a defined microscopic field was counted and statistically analyzed. Consecutive sections of OPSCC were selected for double immunofluorescence analysis to show colocalization of VEGFR2 and CD31 in endothelial cells (EC), to show the localization of VEGFR2 in tumor cells (TC) by double staining for p16INK4A- (in HPV-positive tumors) or for p53 (in HPV-negative tumors). Confocal double immunofluorescence analysis was performed for VEGFR2 and the stem cell marker ALDH1A1.

A total of 56 OPSCC formalin-fixed paraffin-embedded (FFPE) tissue samples having both tumor-containing and adjacent tumor-free regions were available from the Departments of Otorhinolaryngology, Head and Neck Surgery, and Pathology, University of Cologne and were included in this study (Figure 1). Patients were treated between 2011–2013 at the Department of Otorhinolaryngology and Head and Neck Surgery, University of Cologne (Table 1). Primary tonsillar keratinocytes used in cell culture experiments were derived from routine tonsillectomy. The human ethics committee of the University of Cologne approved the procurement of human tumor tissue at surgery and performing research on this material (study number 11–346 for tumor tissue and 18–285 for keratinocytes). Patient material was handled according to the code for proper secondary use of human tissue, and written consent was obtained from all patients.

### 2.2. Tissue Fixation, Embedding and Sectioning

The tissue samples were collected in a fixative containing 4% paraformaldehyde in 0.1 M phosphate buffer saline (PBS) pH 7.4, washed in 0.1 M PBS pH 7.4 at 4 °C and embedded in paraffin. These were then cut with a microtome to obtain 4-μm-thick sections.

### 2.3. DNA Isolation and HPV Typing

The DNA isolation and HPV typing procedures were performed by routine protocols as described previously including GP5+/GP6+ polymerase chain reaction (PCR) followed by direct sequencing and immunohistochemical staining against p16^INK4A^ (Cintec, Roche, Freiburg, Germany), used as a surrogate marker for E7 expression [18].

### 2.4. Immunohistochemistry

Immunohistochemical stainings against the oxidative stress markers NRF2 and AKR1C3 were performed as described previously [15]. In brief, 4 µm-thick sections were deparaffinized and left in 0.01 M citrate buffer (pH 6.0) overnight at 70 °C for antigen retrieval. For detection of NRF2, polyclonal anti-NRF2 antibodies (HPA003097, Sigma-Aldrich, Taufkirchen, Germany; 1:200) together with its corresponding biotinylated secondary antibodies (Abcam, Cambridge, UK; 1:250) were used. AKR1C3 was detected by using monoclonal anti-AKR1C3 antibodies (A6229, Sigma, clone NP6.66.A6, 1:500) and corresponding biotinylated horse anti-mouse antibodies (Vector, Burlingame, CA, USA; 1:250). Antigen retrieval of tissue samples for VEGFR2 staining was carried out by heating at 95 °C in 1mM EDTA buffer (pH 8.0) for 15 min. Slices were incubated with 0.3% H_2_O_2_ in 0.05 M Tris-buffered saline (TBS) for 20 min to inhibit endogenous peroxidase activity. This was followed by treatment with 0.25% Triton-X 100 detergent solution to block non-specific hydrophobic and non-specific ionic interactions. Non-specific immunoglobulin binding sites were blocked using a blocking solution containing 5% normal goat serum (Vector) and 2% bovine serum albumin (Sigma-Aldrich). For the detection of VEGFR2, sections were incubated overnight at 4 °C with rabbit anti-human monoclonal VEGFR2 antibodies (#2479, Cell Signaling Technology, Frankfurt a. M., Germany; clone 55B11, 1:500 in TBS). After incubation for 60 min with biotinylated goat anti-rabbit IgG (Vector, 1:300 in 0.05 M TBS) for antibody detection, subsequently, slides were incubated with avidin-biotin-peroxidase complex (ABC; Vectastain ABC kit, Vector) for 60 min. The peroxidase activity was developed for exactly 15 min with 0.05% 3,3′-diaminobenzidine tetrahydrochloride (DAB; Sigma-Aldrich) in 0.05 M Tris-HCl buffer (pH 7.6), containing 0.01% H_2_O_2_ and 0.01% nickel sulfate. Sections were mounted in xylene-based mounting medium Entellan (Merck, Darmstadt, Germany).

Papillary thyroid carcinoma, liver tissue, melanoma and cervix carcinoma tissues (*n* = 8) served as positive controls (Figure S1). Negative controls were performed without using primary antibodies to test the antibody specificities of the immunohistochemical reagents (Figures S2 and S3).

### 2.5. Double Immunofluorescence Labelling with VEGFR2, CD31, ALDH1A1, p16^INK4A^ and TP53

Double immunofluorescence staining was performed separately in a subseries of consecutive sections using routine protocols published previously [19]. To validate that VEGFR2-positive cells located within blood vessels of HPV-positive and-negative OPSCC are endothelial cells, the samples were incubated with monoclonal anti-human CD31 (a gift from Prof. Dr. M. Koch, Cologne, 1:800) and monoclonal anti-VEGFR2 (1:500; 55B11 Cell Signaling Technology) antibodies. To prove that the VEGFR2 positive epithelial cells are tumor cells and to evaluate differences in the subcellular localization of VEGFR2 in tumor cells of OPSCC, HPV-positive samples were incubated with mouse anti-human polyclonal p16^INK4A^ (1:50; BD Biosciences, Heidelberg, Germany) and VEGFR2 antibodies. HPV-negative samples were incubated with mouse anti-human polyclonal TP53 (Biologo, Kronshagen, Germany, 1:25) and VEGFR2 antibodies. To answer the question of whether individual cells, that are particularly immunoreactive to VEGFR2, represent a subpopulation of cancer stem cells, double staining of VEGFR2 with mouse anti-human monoclonal ALDH1A1 (1:500; H-4 (sc-374076) or B-5 (sc-374149) Santa Cruz, Heidelberg, Germany) was performed in a sub-series of sections (Supplementary Information 1). Subcellular localization patterns were analyzed by confocal microscopy (LSM 710, Carl Zeiss, Oberkochen, Germany).

### 2.6. Densitometric Quantification of Immunohistochemical Signals

The first slice of each sample was stained with hematoxylin and eosin to identify the tumor regions and blood vessels. The following slice of each sample was immunohistochemically incubated with VEGFR2 antibodies. Slides were digitalized in a slide scanner (Leica SCN 400) at 20x magnification. All analyses were carried out in a blinded manner with the bioimage software QuPath (version 0.1.3) [20]. Before the staining intensity was analyzed, the background staining was determined by setting color deconvolution values from a cell-free region of interest for every slide image, so that the program took the background grey values into account when measuring the staining intensities of endothelial or tumor cells. Prior to staining intensity analysis, the regions of interest (ROI) were determined and marked with the software tools. Due to a heterogeneous expression pattern of VEGFR2 within each sample, three different zones of three different staining intensity levels (low, moderate, high) were measured and the respective mean values were used for densitometry in tumor cells. For densitometry in blood vessels, three different vessels from the tumor region and three from the adjacent tumor-free region were selected and the mean intensity values were calculated for both. For the quantification of the blood vessel density, a microscopic field within the tumor region was determined by setting a grid size of 500 mm × 500 mm and the number of VEGFR2-positive vessels was counted within this field.

The quantification of immunohistochemical NRF2- and AKR1C3-stainings was performed as described previously [15]. In brief, tumor cells with positive nuclear staining against NRF2 were considered positive and a lack of staining was negative (Figure S4A,B). For the evaluation of the AKR1C3-staining, an index of staining intensities of the tumor tissue and the adjacent normal squamous epithelium was calculated. Tumors with higher expression intensity in the tumor compared to the surrounding normal tissue were evaluated as positive, those with the same or lower expression intensity in the tumor were evaluated as negative (Figure S4C,D).

### 2.7. Cell Culture and Retroviral Transduction

Primary human tonsillar keratinocytes isolated from routine tonsillectomy were cultivated in RM+medium (consisting of a 3:1 ratio of Dulbecco’s modified Eagle’s medium [DMEM]-F12 with 10% fetal calf serum [FCS], 1% glutamine, 0.4 μg hydrocortisone, 10^−10^ M cholera toxin, 5 μg/mL transferrin, 2 × 10^−11^ M liothyronine, 5 μg/mL insulin, 10 ng/mL epidermal growth factor, 1 × penicillin–streptomycin mixture) [21]. Primary human foreskin keratinocytes were purchased from Lonza (Cologne, Germany, Cat.No. 00192907, Lot.No. 188311) and cultured in Keratinocyte Growth Medium 2 (PromoCell, Heidelberg, Germany). The OPSCC cell line FaDu and the retrovirus packaging cell line PT67 were maintained in DMEM with a 10% FCS and penicillin-streptomycin mixture. All cell lines were cultivated at 37 °C and 6% CO_2_.

Transduction of cells with HPV16-E6, -E7 or -E6E7 coding retroviruses was performed as described previously [21,22,23]. The selection of infected cells was started 2 days later using G418. Positive clones were pooled and expanded.

### 2.8. RNA Isolation, Reverse Transcription and Real-Time Quantitative PCR

To quantify mRNA levels of cellular genes isolated from the above-mentioned human cells from monolayer culture and from fresh frozen OPSCC samples, quantitative reverse transcription-PCR (RT-qPCR) using the LightCycler system (Roche, Mannheim, Germany) was performed as previously described [24]. The primers used for this study were: VEGFA-fw: CCTCCGAAACCATGAACTTT; VEGFA-rev: TTCTTTGGTCTGCATTCACATT; VEGFR1-fw: TTTGGATGAGCAGTGTGAGC; VEGFR1-rev: CGGCACGTAGGTGATTTCTT; VEGFR2-fw: CTCTTGGCCGTGGTGCCTTTG; VEGFR2-rev: GTGTGTTGCTCCTTCTTTCAAC; HPRT1-fw: TGACACTGGCAAAACAATGCA; HPRT1-rev: GGTCCTTTTCACCAGCAAGCT.

### 2.9. Statistics

The sample size was determined before analysis with a power of 90% and a significance level for beta-error ≤ 0.05, including *n* = 35 HPV-positive and *n* = 21 HPV-negative tumor samples. Clinicopathological features were analyzed using cross-tabulations, χ2 test and Fisher’s exact probability test using SPSS 27 software (IBM, Armonk, NY, USA). The overall survival was calculated using the Kaplan–Meier algorithm for incomplete observations. Outcomes were measured from the time of diagnosis to the last day the patient was alive (censored data) or died for any reason (uncensored data). The log-rank (Mantel-Cox) test was used to perform a univariate analysis of the different variables. RT-qPCR data were analyzed with GraphPad Prism 8 (GraphPad Software, La Jolla California, USA) using ANOVA. The staining intensities of tumor cells, blood vessels or vessel density were analyzed using the Wilcoxon test for dependent non-normally distributed groups, the Mann–Whitney U test for independent non-normally distributed groups and the t-test for independent normally distributed groups as indicated. Results at a significance level of p ≤ 0.05 in two-sided tests were considered statistically significant. All data from RT-qPCRs were expressed as mean ± SD. Statistical significance was determined with unpaired two-tailed Student’s t-test. *, *p* < 0.05; **, *p* < 0.01; ***, *p* < 0.001.

## 3. Results

### 3.1. Characterization of VEGFR2 Expression in Blood Vessels of HPV-Positive and HPV-Negative OPSCC

Since ROS and OS were previously demonstrated to have a strong effect on the expression of VEGF/VEGFR2, we aimed at characterizing whether differences in the expression pattern of VEGFR2 exist in 56 OPSCC with known HPV-status and OS signatures [13,25]. We, therefore, determined VEGFR2 levels on blood vessels from tumor-free and tumor-containing regions. Furthermore, we aimed at detecting differences in the vessel density of HPV-positive versus HPV-negative OPSCC.

**Figure 2:**
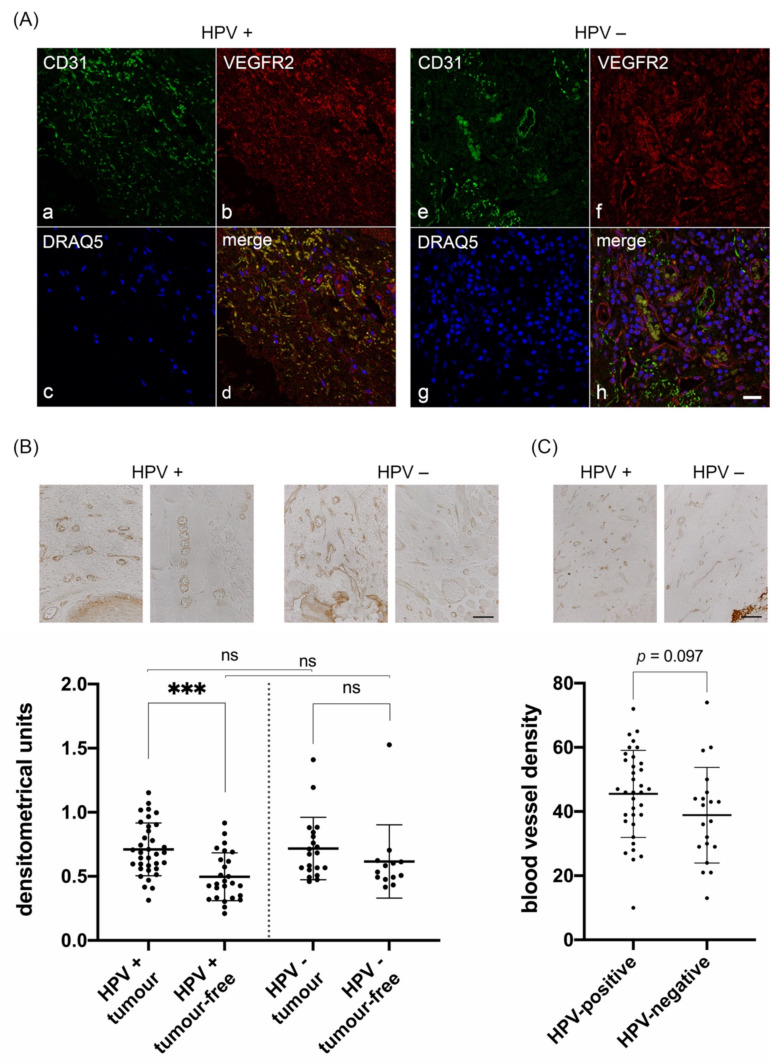
(**A**) Colocalization analysis of VEGFR2 with CD31 (endothelial cell marker) by immunofluorescence labelling of OPSCC. Antibody stainings were visualized by confocal microscopy. Colocalization of CD31 (**a**) with VEGFR2 (**b**) revealed that VEGFR2 is expressed in numerous capillaries of HPV-positive OPSCC (**d**). Colocalization of CD31 (**e**) with VEGFR2 (**f**) revealed that VEGFR2 is only occasionally present in capillaries of HPV-negative tumors (**h**). Cell nuclei were stained with DRAQ5 (**c,g**). Scale bar: A-H 20 µm. (**B**) Representative immunohistochemical staining and corresponding staining intensity analysis of VEGFR2 expression in blood vessels of tumor containing and adjacent tumor-free regions in (left) HPV-positive and (right) HPV-negative OPSCC. HPV-positive tumor regions (M = 0.657 DU; SD = 0.178 DU), adjacent tumor-free regions (M = 0.497; SD = 0.187), (***, *p* < 0.0001); HPV-negative tumor regions (M = 0.675; SD = 0.251), tumor-free regions (M = 0.616; SD = 0.286), (ns, *p* = 0.107); HPV-positive and -negative tumor regions (ns, *p* = 0.740); HPV-positive and-negative tumor-free regions (ns, *p* = 0.129). (**C**) Blood vessel density per viewing field of VEGFR2-immunoreactive capillaries in HPV-positive and HPV-negative OPSCC. Vascular density of HPV-positive (M = 45.51; SD = 13.574) and HPV-negative OPSCC (M = 38.85; SD = 14.901), (ns, *p* = 0.097). (DU = densitometrical units, M = mean, SD = standard deviation, ns = not significant, scale bars: A 50 μm; B 100 μm).

To prove that the VEGFR2-positive cells are blood vessel lining endothelial cells, we performed double immunofluorescence staining with the endothelial cell marker CD31 together with VEGFR2. In most capillaries of the HPV-positive samples, endothelial cells showed strong colocalization of CD31 with VEGFR2. In HPV-negative sections, however, a sparse colocalization of CD31-positive endothelial cells with VEGFR2 was observed (Figure 2A).

Next, we performed immunohistochemical staining to analyze differences in the expression level of VEGR2 in blood vessels related to the HPV-status of the tumor. In both HPV-positive and -negative tumors, blood vessels were positive for VEGFR2 immunostaining, however, with obvious differences in the staining intensities (Figure 2B).

We next determined the staining intensity of VEGFR2 by densitometrical analysis of representative viewing fields of three blood vessels each in tumor regions and tumor-free regions for comparison within the same sections (in densitometrical units (= DU)). Interestingly, in the subgroup of HPV-positive tumors, we observed significantly stronger staining intensities of blood vessels in tumor regions compared to tumor-free regions (Figure 2B, left; *p* < 0.0001). In contrast, in the HPV-negative group, the difference in staining intensities of blood vessels between tumor regions and tumor-free regions was not significant (Figure 2B, right; *p* = 0.107). Also, when comparing blood vessels of HPV-positive and -negative tumor or tumor-free regions, differences in VEGFR2 staining intensities were not significant either (*p* = 0.740 and *p* = 0.129, respectively).

To further analyze whether VEGFR2 mediated angiogenesis might be generally increased in HPV-positive compared to HPV-negative OPSCC, the number of VEGFR2-immunoreactive capillaries per viewing field was analyzed. However, comparing both groups, only a trend, but no significant increase in the number of vessels could be observed (Figure 2C; *p* = 0.097).

### 3.2. Analysis of VEGFR2 Staining Intensity in Tumor Cells of HPV-Positive and HPV-Negative OPSCC

Since we observed that VEGFR2 expression was not only restricted to blood vessels but could also be detected within tumor cells, we additionally analyzed whether the HPV-status influences VEGFR2 immunoreactivity within tumors. Regardless of the HPV-status, we observed a general heterogeneity of staining intensities within each tumor section with areas of low, medium and high VEGFR2 expression.

**Figure 3:**
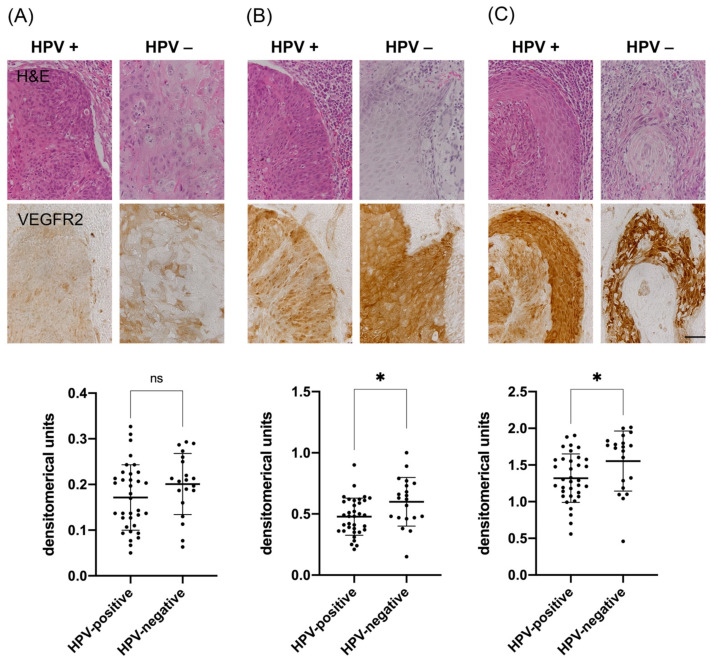
Analysis of VEGFR2 staining intensity in tumor cells of HPV-positive and HPV-negative OPSCC. Representative immunohistochemical VEGFR2 staining images (A = low, B = medium and C = high staining intensity), as well as corresponding H&E stain images of consecutive sections. (**A**) Staining intensities of low VEGFR2 expressing HPV-positive (M = 0.172 DU; SD = 0.072 DU) and HPV-negative (M = 0.201 DU; SD = 0.067 DU) tumor cells, *p* = 0.140. (**B**) Moderate expression levels of VEGFR2-positive tumor cells in HPV-positive (M = 0.478; SD = 0.152) vs. HPV-negative samples (M = 0.600; SD = 0.199), *, *p* = 0.014. (**C**) High expression levels of VEGFR2-positive tumor cells in HPV-negative (M = 1.714; SD = 0.260) and HPV-positive specimens (M = 1.382; SD = 0.320), *, *p* = 0.012. (DU = densitometrical units, M = mean, SD = standard deviation, ns = not significant, scale bar 50 μm).

To determine these expression differences, areas of low (median 0.2 DU (CI 0.13–0.23)), medium (median 0.49 DU (CI 0.38–0.63)) and high staining intensities (median 1.45 DU (CI 1.12–1.75)) were measured by densitometry for each tumor sample. In general, VEGFR2 expression was found to be higher in HPV-negative tumors. This difference was particularly significant for VEGFR2 expression levels at high-intensity (Figure 3C; *p* = 0.012) and at medium intensity (Figure 3B; *p* = 0.014). In regions with low VEGFR2 expression, this difference was not significant but again showed a trend toward higher expression in HPV-negative tumors (Figure 3A; *p* = 0.140).

In order to clarify whether the VEGFR1, VEGFR2 and VEGFA genes are transcriptional targets of HPV16 oncogenes, we quantified their mRNA expression levels by RT-qPCR in monolayer cultures of FaDu cells, primary tonsillar keratinocytes as well as primary foreskin keratinocytes. No strong effects could be measured in all keratinocyte types for VEGFR1 and VEGFA (Figure S5A–C). VEGFR2 could not be quantified at all in cell culture. However, in RNAs, isolated from fresh OPSCC samples, the mRNA expressions of VEGFR1 and VEGFR2 were significantly higher in HPV-positive samples than in HPV-negative samples. (Figure S5D).

**Figure 4:**
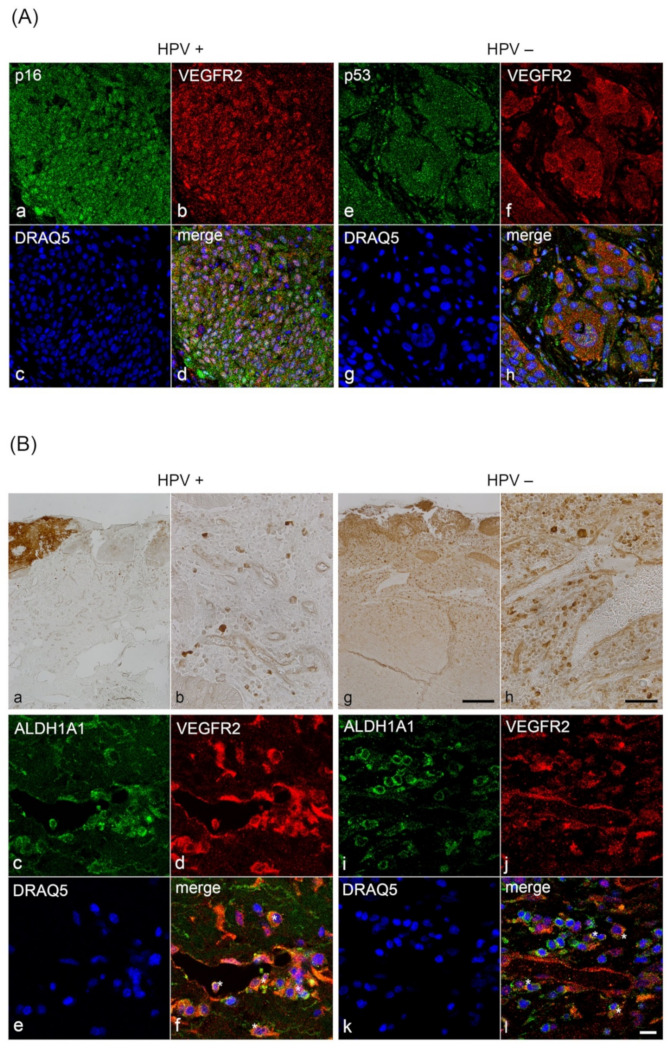
(**A**) Colocalization analysis of VEGFR2 with p16^INK4A^ (tumor cell marker of HPV-positive tumors) and VEGFR2 with p53 (tumor cell marker of HPV-negative tumors) by immunofluorescence labeling of OPSCC. Antibody stainings were visualized by confocal microscopy. Colocalization of p16^INK4A^ (**a**) with VEGFR2 (**b**) was detected in the cytoplasm and cell nuclei of HPV-positive tumor cells (**d**). Colocalization of p53 (**e**) with VEGFR2 (**f**) was identified in the cytoplasm and cell nuclei of HPV-negative tumor cells. Note, that VEGFR2 was detected in numerous tumor cell nuclei of HPV-positive OPSCC (**d**) compared to HPV-negative OPSCC (**h**). Tumor cell nuclei were stained with DRAQ5 (**c**,**g**). Scale bar: 20 μm. (**B**) Colocalization analysis of VEGFR2 with ALDH1A1 (CSC marker) by immunofluorescence labeling of OPSCC. Immunohistochemical staining against VEGFR2 of a representative consecutive HPV-positive (**a**–**f**) and HPV-negative tumor section (**g**–**l**). (**a**,**g**) Overview and (**b**,**h**) details. A subpopulation of tumor cells with strong VEGFR2 immunoreactivity can be observed. Colocalization of VEGFR2 (**d**,**j**) with ALDH1A1 (**f**,**l**) was detected only in a subpopulation of tumor cells and at the subcellular level mainly in the cytoplasm (asterisks). The cells were distributed around the blood vessels (**f**,**l**). In HPV-positive OPSCC, some migrating cells were detected at the blood vessel wall and one cell is visible intravasally, while others were recognized in the fibrous tissue (**f**). Cell nuclei were stained with DRAQ5 (**e**,**k**). Scale bars: (**a**,**g**) 200 μm; (**b**,**h**) 50 μm; (**c**–**f**), (**i**–**l**) 20 μm.

To prove the keratinocytic origin of VEGFR2-positive tumor cells and to evaluate differences in the subcellular localization of VEGFR2, we performed double immunofluorescence analyses with antibodies against p16^INK4A^ to detect HPV-positive tumor cells and TP53 to detect HPV-negative tumor cells. In both tumor entities, cytoplasmic and nuclear VEGFR2-immunoreactivity was detected. However, nuclear expression of VEGFR2 in HPV-positive OPSCC samples was found to be stronger (Figure 4A). Additionally, strong VEGFR2-positive cells, which were diffusely distributed in the lamina propria and around capillaries were also found in both tumor entities. However, these cells were detected more frequently in HPV-positive (*n* = 16/35) than in HPV-negative (*n*= 7/21) tumors.

Based on the fact that VEGFR2 positive cells were localized at sites typical for the localization of cancer stem cells (CSCs), we examined whether these cells might show CSCs characteristics [6]. Therefore, we performed double immunofluorescence staining with VEGFR2 and the CSC-marker ALDH1A1 [26]. Distinct cells showed colocalization of VEGFR2 with ALDH1A1, which was paralleled by high cytoplasmic ALDH1A1 expression levels. Cells with a distinct colocalization pattern were predominantly found next to blood vessels. These colocalization signals were frequently observed in HPV-positive OPSCC, however, we sparsely detected them in HPV-negative OPSCC (Figure 4B).

### 3.3. Correlation of VEGFR2, NRF2 and AKR1C3 with Clinicopathological Data

We recently demonstrated that the oxidative stress markers NRF2 and AKR1C3 are overexpressed in both subgroups of HPV-positive and HPV-negative OPSCC and correlate with unfavorable survival [15]. As ROS and OS were demonstrated to have a strong effect on the expression of VEGF/VEGFR2, we performed immunohistochemical staining against NRF2 and AKR1C3 to prove the occurrence of OS in the tumor tissue (Figure S4) [13,25]. By correlating the immunohistochemical results with clinicopathological data, we demonstrated a highly significant correlation of NRF2^high^ and AKR1C3^high^ tumors with worse overall survival (OS) (*p* < 0.0001 and *p* < 0.001, respectively) (Table 1 and Table S1). We also separately correlated VEGFR2, NRF2 and AKR1C3 staining in HPV-positive and HPV-negative tumors in relation to survival. These analyses revealed that particularly high VEGFR2 expression in HPV-positive tumors is associated with an unfavorable prognosis (*p* = 0.013) (Figure S6 and Table S1). High T-stage correlated with worse OS (*p* = 0.049) in all cases (Table S1). However, T-stage did not reach significance in separate correlations of both HPV-positive and-negative subgroups (Figure S7). There was a strong association for VEGFR2 protein expression with HPV-status (Table 1). Furthermore, HPV-status was associated with parameters such as a higher blood vessel density and a higher proportion of non-smokers to smokers (Table 1).

## 4. Discussion

Autocrine VEGF signaling can contribute to tumor initiation and progression and is regulated by the receptor tyrosine kinase VEGFR2 in several tumors including HNSCC [6,10]. Although it is known that VEGFR2 is generally overexpressed in blood vessels supplying HNSCC, it remains to be determined whether HPV exerts specific effects on VEGFR2 expression and thus possibly on the regulation of vascularization [9,10]. Studies on cervical tumors and in vitro studies using HPV-positive cervical cancer cell lines indicated such a possibility [11,12,27]. It is speculated that HPV generally contributes to the vascularization of tissues by upregulating VEGF/VEGFR signaling to support the high energy demands of infected cells, which in turn also promotes tumor growth and malignant transformation [27,28]. In addition, a variety of tumor cells, including HNSCC, are known to express VEGF and VEGFR2 at high levels so that they can promote tumor growth, invasion and survival by autocrine signaling responses [6,10,29]. However, previous studies on HNSCC have not focused specifically on OPSCC and HPV-status has not been considered [9].

We, therefore, analyzed the expression patterns of VEGFR2 both in blood vessels of tumor regions and tumor-free regions and in tumor cells of HPV-positive and -negative OPSCC by means of densitometric analysis. Since oxidative stress (OS) is known to induce VEGF expression and we previously showed that both HPV-positive and -negative OPSCC present with subgroups overexpressing OS signatures going along with poor prognosis, NRF2 expression and analysis of its target gene AKR1C3 serving as a read-out for activated OS signatures was included in this study [30,31]. Differences in VEGFR2 expression between HPV-positive and -negative OPSCC were detectable in both blood vessels and tumor tissue, suggesting that depending on HPV status, VEGFR2 signaling plays a crucial role in OPSCC progression.

Our results strongly imply that VEGFR2 may be upregulated in endothelial cells of blood vessels supplying nutrients to HPV-positive tumor regions and that this is not the case in HPV-negative OPSCC. Therefore, our observation might suggest that angiogenesis may be upregulated under the influence of HPV by regulating the expression of VEGFR2 in blood vessels. However, further studies have to clarify by which mechanisms HPV-positive tumor cells might regulate increased VEGFR2 expression in surrounding tumor blood vessels.

This hypothesis is furthermore supported by the observation that HPV-positive OPSCC tends to have a higher density of VEGFR2-expressing blood vessels compared to HPV-negative tumors (Figure 2). While this analysis did not reach significance, the absolute number of tumors with a high density of VEGFR2-expressing blood vessels, however, proved to be significantly higher (Table 1). This key observation is supported by xenograft models in which HPV-positive and -negative cells were incubated under hypoxic conditions and then applied to nude mice [32]. Those mice that received HPV-positive tumor cells showed a higher density of neo-blood vessels, which resulted in improved blood supply and thus less hypoxic tumor areas, which was paralleled by lower mRNA expression of hypoxia-responsive genes such as HIF-1α, GLUT-1 and VEGF-A. Moreover, in that study, tumor samples were analyzed by immunohistochemical detection of neo-blood vessels using the vascular endothelial cell proliferation marker CD105. This led to the observation of HPV-positive OPSCC having higher numbers of blood vessels.

We, therefore, considered analyzing the activation status of VEGFR2 to detect only angiogenically active blood vessels by immunohistochemical staining with antibodies directed against phosphorylated VEGFR2 at Tyr 951, as this modification plays a significant role in tumor angiogenesis and growth [33]. However, we could not detect specific immunoreactivity using the only available antibody suitable for immunohistochemistry thus far (monoclonal Phospho-VEGFR2 (Tyr951) (15D2); #4991 Cell Signaling; data not shown).

The expression of CD31 in endothelial cells modulates cell adhesion, endothelial cell migration and angiogenesis [30]. However, CD31 only gives a static representation of vessel density, whereas VEGFR2 expression may better reflect the physiological stimulus for endothelial growth [31]. In HPV-negative tumors, VEGFR2 and CD31 only partially colocalized in the endothelium of blood vessels and capillaries. The strong colocalization of CD31 and VEGFR2 in blood vessels and capillaries of HPV-positive OPSCC, however, indicates that HPV induces a highly upregulated angiogenic activity, pointing to significant differences in angiogenesis based on HPV-status [34,35]. This upregulated angiogenic activity may result in an improved response to radiochemotherapy, as individual tumor cells may be more accessible by the bloodstream, leading to a more favorable prognosis compared to HPV-negative OPSCC [36,37]. Mechanistically, increased perfusion delivers oxygen that promotes ROS/free radicals essential for the induction of radiation-induced DNA damage upon radiotherapy thus making cells more accessible for the influx of chemotherapeutic agents.

On the other hand, an improved radiation response may also be achieved by blocking VEGFR2 through anti-VEGF therapy [38,39]. Typically, epithelial tumors can respond to radiotherapy with growth factor-driven revascularization, including increased VEGFR2 expression, which may be prevented by anti-VEGF therapy that inhibits revascularization. This, in turn, would lead to increased blood flow and thus a better oxygen supply, resulting in increased ROS formation during radiation therapy and increased flooding of the chemotherapy to the already existing tumor cells. Due to the lack of new blood vessel formation, on the other hand, the formation of new tumor tissue is prevented [37,38,39]. In this study, upregulation of VEGFR2 and the OS marker AKR1C3 was associated with an unfavorable prognosis in HPV-positive OPSCC.

In addition, we could show that VEGFR2 is also expressed in tumor cells. Both HPV-positive and -negative OPSCC presented with a heterogeneous expression pattern including low, moderate and high VEGFR2-immunoreactivity. This may suggest that aberrant VEGFR2 expression in tumor cells, together with its downstream signaling pathways, may be involved in other besides angiogenesis. However, mutations in VEGFR2 do not seem to be of relevance, as they only show low alteration rates in HNSCC (2.3% in the TCGA cohort, cBioPortal, data not shown [40,41]). Therefore, other mechanisms like varying differentiation states of tumor cells within one tumor, or areas that have a need for improved nutrients and oxygen supply compared to other regions, might correlate with increased VEGFR2 expression levels.

Densitometric analysis showed a significantly higher expression of VEGFR2 in HPV-negative tumor cells. This may suggest that the differential expression of VEGFR2 between HPV-positive and -negative tumor cells exhibit crucial cell biological differences.

Several oncogenes such as the epidermal growth factor receptor (EGFR) and oncogenic transcription factors such as c-myc are capable of upregulating VEGF expression. Furthermore, wild-type TP53 indirectly represses VEGF [42]. However, HPV16-E6 seems to induce VEGF expression independently of TP53 inactivation, using the SP1 transcription factor for E6-mediated induction of the VEGF promoter [11]. This could also be supported by our observation that VEGFR2 was frequently translocated into the nuclei of HPV-positive but less frequently into the nuclei of HPV-negative tumor cells. It is known that VEGFR2 is translocated to the nucleus of neoplastic cells upon phosphorylation and that VEGFR2 therein may interact with transcription factors such as SP1 to regulate gene transcription [43,44]. Biologically, this might be a self-enhancing mechanism in response to hypoxia and/or OS. In vitro studies using HeLa cells showed that the fraction of nuclear-positive cells increased due to hypoxic stimulation [43].

Degradation of VEGFR2 is furthermore mediated by the recruitment of the E3 ubiquitin ligase β-Trcp1 followed by polyubiquitination and delivery to the proteasome [45]. β-Trcp1 is upregulated by HPV16-E7 expression in vitro [46]. Moreover, β-Trcp1 is also involved in the regulation of HIF1α the WNT/β-Catenin and the PI3K/AKT pathway [47,48]. β-Trcp1 also provides an alternative way to regulate the NRF2/OS pathway for proteasomal degradation via Keap1/Cul3 [49]. Therefore, additional side effects may exist, especially considering that all these signaling pathways often present with alterations especially in HPV-negative OPSCC.

Noteworthily, a subpopulation of tumor cells presented with high VEGFR2 expression levels. Based on their localization in a perivascular niche adjacent to endothelial cells, it may be suggested that these cells are CSCs. It is known that the amount of this stem cell population and their self-renewal can be regulated by autocrine VEGF/VEGFR2 signaling [6,50]. Double immunostaining with the tumor stem cell marker ALDH1A1 maintaining CSC properties indeed showed colocalization of VEGFR2 with ALDH1A1. This suggests that these cells might be CSCs localized in the proposed perivascular niche [26].

In line with studies analyzing CSCs in the comparison of HPV-positive and -negative OPSCC, we observed a higher number of ALDH1A1^+^/VEGFR2^+^ in HPV-positive OPSCC [51,52]. We recently showed that HPV16 is capable of modifying the phenotype of infected CSCs by increasing the pool of migratory CSCs through the expression of HPV16-E6E7 in vitro and in HPV-positive OPSCC [22]. Furthermore, autocrine VEGF/VEGFR2 signaling enhances tumor invasion and survival by promoting processes crucial for CSCs like dedifferentiation and an epithelial-mesenchymal transition phenotype [6]. Furthermore, VEGFR2 can regulate epithelial tumor stem cell migration [53]. Taken together, the higher number of ALDH1A1 and VEGFR2 colocalizing tumor cells in HPV-positive OPSCC might indicate that these tumors are associated with a higher number of migratory CSCs.

## 5. Conclusions

To analyze VEGFR2 expression patterns in HPV-positive and-negative OPSCC, we performed quantitative immunohistochemical and immunofluorescence labeling of formalin-fixed, paraffin-embedded (FFPE) samples with known HPV-status. VEGFR2-staining intensities in blood vessels of tumor-containing and tumor-free regions, as well as in tumor cells, were quantified and compared between HPV-associated and HPV-negative OPSCC.

**Figure 5:**
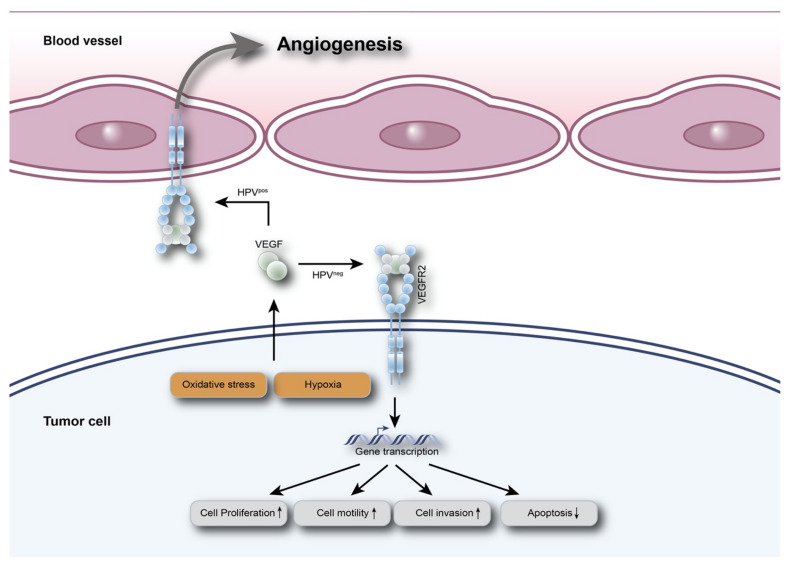
Schematic presentation of the two suggested HPV-status-dependent VEGFR2 signal pathways in OPSCC. Depending on HPV and possibly triggered by hypoxia and/or oxidative stress, VEGFR2 expression is upregulated in tumor blood vessels of HPV-positive OPSCC, which may be associated with increased angiogenesis. This is not observed in HPV-negative OPSCC; instead, VEGFR2 is significantly stronger expressed in the tumor cells themselves, which may lead to increased activation of tumor cell proliferation, migration, invasion and reduced apoptosis. The increased tumor cell activity may also be correlated to the tendency of lower blood vessel count in HPV-negative OPSCC due to hypoxia. Figure modified from [10].

In conclusion, we identified a distinct molecular protein expression profile of VEGFR2 in HPV-positive and HPV-negative OPSCC. In the HPV-positive group, we observed significant differences in VEGFR2 expression levels between blood vessels of tumor regions compared to tumor-free regions. In contrast, HPV-negative OPSCC presented with significantly higher VEGFR2 expression levels in tumor cells. Based on this observation, two different HPV-status-dependent phenotypes of VEGFR2 signaling may exist, possibly triggered by hypoxia and/or oxidative stress (Figure 5). Future studies should focus on unraveling the molecular basis of mechanisms involved in the differential regulation of VEGFR2 expression between HPV-positive and -negative OPSCC in tumor-supporting blood vessels and tumor cells. Such studies could prove to be of pivotal importance for the patient outcome when anti-VEGF therapies in the treatment of OPSCC are considered.

Furthermore, our data indicate that VEGFR2 may play a regulatory role in CSCs of HPV-positive OPSCC. CSCs are thought to be responsible for treatment failure in anti-cancer therapy. Experimental validation of the regulatory role of VEGFR2 in stem cell migration and dedifferentiation, especially in CSCs of HPV-positive OPSCC, will further contribute to the understanding of unfavorable development of (distant) metastases and recurrence.

## Figures and Tables

**Table 1:** Summary of clinicopathological features of patients analyzed in this study.

Clinico Pathological Feature	Total		HPV—Status					VEGFR2—Staining					NRF2—Staining				
			HPV—positive		HPV—negative			VEGFR+		VEGFR2−			NRF2+		NRF2−		
	n	%	n	%	n	%	*X* ^2^	n	%	n	%	*X* ^2^	n	%	n	%	*X* ^2^
Mean age (years)	60.2	–	59.9	–	60.5	–	0.838	59.2	–	61.0	–	0.517	62.9	–	58.8	–	0.177
Gender																	
Male	43	76.8	26	46.8	17	30.0		23	41.1	19	33.9		17	30.3	26	46.4	
Female	13	23.2	9	16.1	4	7.1	0.747	2	3.6	11	19.6	**0.023**	2	3.6	11	19.6	0.098
T classification																	
pT1 and pT2	30	53.6	20	35.7	10	17.9		13	23.6	16	29.1		8	14.3	22	39.3	
pT3 and pT4	26	46.4	15	26.8	11	19.6	0.584	12	21.8	14	25.5	1.00	11	19.6	15	26.8	0.265
N classification																	
pN0	14	25.0	9	16.1	5	8.9		4	7.3	9	16.4		4	7.1	10	17.9	
pN1-3	42	75.0	26	46.4	16	28.6	1.00	21	38.2	21	38.2	0.341	15	26.8	27	48.2	0.751
M classification																	
pM0	54	98.2	35	63.6	19	34.5		24	44.4	29	53.7		18	32.7	36	65.5	
pM1	1	1.8	0	0.0	1	1.8	0.364	0	0.0	1	1.9	1.00	1	1.8	0	0.0	0.345
Death																	
Yes	16	28.6	9	16.1	7	12.5		6	10.9	10	18.2		12	21.4	4	7.1	
No	40	71.4	26	46.4	14	25.0	0.557	24	43.6	15	27.3	0.140	7	12.5	33	58.9	**<0.0001**
HPV-status																	
Negative	21	37.5						13	23.6	7	12.7		7	12.5	14	25.0	
Positive	35	62.5						12	21.8	23	41.8	**0.048**	12	21.4	23	41.1	1.00
NRF2-staining																	
Negative	36	65.5	23	41.1	14	25.0		14	25.5	22	40.0						
Positive	19	34.5	12	21.4	7	12.5	1.00	11	20.0	8	14.5	0.256					
AKR1C3-staining																	
Yes	38	67.9	25	44.6	13	23.2		15	27.3	22	40.0		14	25.5	22	40.0	
No	18	32.1	10	17.9	8	14.3	0.558	10	18.2	8	14.5	0.389	11	20.0	8	14.5	0.256
Blood vessel density																	
Low	30	54.5	15	27.3	15	27.3		12	21.8	18	32.7		7	12.7	23	41.8	
High	25	45.5	20	36.4	5	9.1	**0.027**	13	23.6	12	21.8	0.424	12	21.8	13	23.6	0.087
Smoking																	
Never smoked	13	23.2	12	21.4	1	1.8		3	5.5	10	18.2		3	5.4	10	17.9	
Smoker	43	76.8	23	41.1	20	35.7	**0.020**	22	40.0	20	36.4	0.110	16	28.6	27	48.2	0.507
Alcohol																	
No or < 1 glass/day	43	78.2	30	53.6	14	25.0		17	30.9	26	47.3		17	30.4	27	48.2	
Active, > 1 glass/day	12	21.8	5	8.9	7	12.5	0.108	8	14.5	4	7.3	0.114	2	3.6	10	17.9	0.189

*n* = Number of patients. *X*^2^: Chi-Square test for significance. For mean age, Anova is used to measure significance. Significant values are highlighted in bold.

## Data Availability

The data presented in this study are available on request from the corresponding authors. The data are not publicly available due to ethical restrictions.

## Supplementary Materials

The following are available online at https://www.mdpi.com/article/10.3390/cancers13205221/s1, Supplementary Information 1, Figure S1: Immunohistochemical positive controls of VEGFR2 expression in tissue samples of liver, cervix squamous cell carcinoma, melanoma, papillary thyroid carcinoma Figure S2: Immunohistochemical controls of the secondary antibodies and the detection system, Figure S3: Control of the confocal double immunofluorescence detection system, Figure S4: Representative immunohistochemical stainings of NRF2 and AKR1C3 in tumor tissue samples, Figure S5: MRNA expression of VEGFA, VEGFR1 and VEGFR2 were measured using reverse transcribed total cellular RNA from FaDu cells, primary tonsil keratinocytes, primary foreskin keratinocytes and HPV-negative or -positive OPSCC by RT-qPCR and normalized to HPRT1 mRNA levels, Figure S6: Univariate survival analysis for VEGFR2, NRF2 and AKR1C3 expression-status separated by HPV-status, Figure S7: Univariate survival analysis for T-stage and N-stage separated by HPV-status, Table S1: Univariate survival analysis.

## References

[1] Chaturvedi A.K., Engels E.A., Pfeiffer R.M., Hernandez B.Y., Xiao W., Kim E., Jiang B., Goodman M.T., Sibug-Saber M., Cozen W. (2011). Human papillomavirus and rising oropharyngeal cancer incidence in the United States. J. Clin. Oncol..

[2] CDC Cancers Associated with Human Papillomavirus, United States—2012–2016. USCS Data Brief, no 10. https://www.cdc.gov/cancer/uscs/about/data-briefs/no10-hpv-assoc-cancers-UnitedStates-2012-2016.htm.

[3] Leemans C.R., Snijders P.J.F., Brakenhoff R.H. (2018). The molecular landscape of head and neck cancer. Nat. Rev. Cancer.

[4] Hoffmann M., Tribius S. (2018). HPV and oropharyngeal squamous cell cancer in the 8th edition of the TNM classification. Laryngorhinootologie.

[5] Simons M., Gordon E., Claesson-Welsh L. (2016). Mechanisms and regulation of endothelial VEGF receptor signalling. Nat. Rev. Molec. Cell Biol..

[6] Goel H.L., Mercurio A.M. (2013). VEGF targets the tumour cell. Nat. Rev. Cancer.

[7] Karaman S., Leppänen V.-M., Alitalo K. (2018). Vascular endothelial growth factor signaling in development and disease. Development.

[8] Kowanetz M., Ferrara N. (2006). Vascular endothelial growth factor signaling pathways: Therapeutic perspective. Clin. Cancer Res..

[9] Neuchrist C., Erovic B.M., Handisurya A., Steiner G.E., Rockwell P., Gedlicka C., Burian M. (2001). Vascular endothelial growth factor receptor 2 (VEGFR2) expression in squamous cell carcinomas of the head and neck. Laryngoscope.

[10] Christopoulos A., Ahn S.M., Klein J.D., Kim S. (2011). Biology of vascular endothelial growth factor and its receptors in head and neck cancer: Beyond angiogenesis. Head Neck.

[11] López-Ocejo O., Viloria-Petit A., Bequet-Romero M., Mukhopadhyay D., Rak J., Kerbel R.S. (2000). Oncogenes and tumor angiogenesis: The HPV-16 E6 oncoprotein activates the vascular endothelial growth factor (VEGF) gene promoter in a p53 independent manner. Oncogene.

[12] Tang X., Zhang Q., Nishitani J., Brown J., Shi S., Le A.D. (2007). Overexpression of human papillomavirus type 16 oncoproteins enhances hypoxia-inducible factor 1 alpha protein accumulation and vascular endothelial growth factor expression in human cervical carcinoma cells. Clin. Cancer Res..

[13] Zimta A.A., Cenariu D., Irimie A., Magdo L., Nabavi S.M., Atanasov A.G., Berindan-Neagoe I. (2019). The Role of Nrf2 Activity in Cancer Development and Progression. Cancers.

[14] Penning T.M. (2017). Aldo-keto reductase regulation by the Nrf2 system: Implications for stress response, chemotherapy drug resistance, and carcinogenesis. Chem. Res. Toxicol..

[15] Huebbers C.U., Verhees F., Poluschkin L., Olthof N.C., Kolligs J., Siefer O.G., Henfling M., Ramaekers F.C.S., Preuss S.F., Beutner D. (2019). Upregulation of AKR1C1 and AKR1C3 expression in OPSCC with integrated HPV16 and HPV-negative tumors is an indicator of poor prognosis. Int. J. Cancer.

[16] Cancer Genome Atlas Network (2015). Comprehensive genomic characterization of head and neck squamous cell carcinomas. Nature.

[17] Martinez V.D., Vucic E.A., Thu K.L., Pikor L.A., Lam S., Lam W.L. (2015). Disruption of KEAP1/CUL3/RBX1 E3-ubiquitin ligase complex components by multiple genetic mechanisms: Association with poor prognosis in head and neck cancer. Head Neck.

[18] Meyer M.F., Huebbers C.U., Siefer O.G., Vent J., Engbert I., Eslick G.D., Valter M., Klussmann J.P., Preuss S.F. (2014). Prevalence and risk factors for oral human papillomavirus infection in 129 women screened for cervical HPV infection. Oral Oncol..

[19] Korkmaz Y., Roggendorf H.C., Siefer O.G., Seehawer J., Imhof T., Plomann M., Bloch W., Friebe A., Huebbers C.U. (2018). Downregulation of the α1- and β1-subunit of sGC in arterial smooth muscle cells of OPSCC Is HPV-independent. J. Dent. Res..

[20] Bankhead P., Loughrey M.B., Fernández J.A., Dombrowski Y., McArt D.G., Dunne P.D., McQuaid S., Gray R.T., Murray L.J., Coleman H.G. (2017). QuPath: Open source software for digital pathology image analysis. Sci. Rep..

[21] Hufbauer M., Biddle A., Borgogna C., Gariglio M., Doorbar J., Storey A., Pfister H., Mackenzie I., Akgul B. (2013). Expression of betapapillomavirus oncogenes increases the number of keratinocytes with stem cell-like properties. J. Virol..

[22] Hufbauer M., Maltseva M., Meinrath J., Lechner A., Beutner D., Huebbers C.U., Akgul B. (2018). HPV16 increases the number of migratory cancer stem cells and modulates their miRNA expression profile in oropharyngeal cancer. Int. J. Cancer.

[23] Halbert C.L., Demers G.W., Galloway D.A. (1992). The E6 and E7 genes of human papillomavirus type 6 have weak immortalizing activity in human epithelial cells. J. Virol..

[24] Hufbauer M., Cooke J., van der Horst G.T., Pfister H., Storey A., Akgul B. (2015). Human papillomavirus mediated inhibition of DNA damage sensing and repair drives skin carcinogenesis. Mol. Cancer.

[25] Ushio-Fukai M., Nakamura Y. (2008). Reactive oxygen species and angiogenesis: NADPH oxidase as target for cancer therapy. Cancer Lett..

[26] Qian X., Wagner S., Ma C., Coordes A., Gekeler J., Klussmann J.P., Hummel M., Kaufmann A.M., Albers A.E. (2014). Prognostic significance of ALDH1A1-positive cancer stem cells in patients with locally advanced, metastasized head and neck squamous cell carcinoma. J. Cancer Res. Clin. Oncol..

[27] Pinheiro C., Garcia E.A., Morais-Santos F., Moreira M.A., Almeida F.M., Jube L.F., Queiroz G.S., Paula E.C., Andreoli M.A., Villa L.L. (2015). Reprogramming energy metabolism and inducing angiogenesis: Co-expression of monocarboxylate transporters with VEGF family members in cervical adenocarcinomas. BMC Cancer.

[28] Chandel V., Raj S., Kumar P., Gupta S., Dhasmana A., Kesari K.K., Ruokolainen J., Mehra P., Das B.C., Kamal M.A. (2020). Metabolic regulation in HPV associated head and neck squamous cell carcinoma. Life Sci..

[29] Lalla R.V., Boisoneau D.S., Spiro J.D., Kreutzer D.L. (2003). Expression of vascular endothelial growth factor receptors on tumor cells in head and neck squamous cell carcinoma. Arch. Otolaryngol. Head Neck Surg..

[30] Newman P.J. (1997). The biology of PECAM-1. J. Clin. Investig..

[31] Bosmuller H., Pfefferle V., Bittar Z., Scheble V., Horger M., Sipos B., Fend F. (2018). Microvessel density and angiogenesis in primary hepatic malignancies: Differential expression of CD31 and VEGFR-2 in hepatocellular carcinoma and intrahepatic cholangiocarcinoma. Pathol. Res. Pract..

[32] Hanns E., Job S., Coliat P., Wasylyk C., Ramolu L., Pencreach E., Suarez-Carmona M., Herfs M., Ledrappier S., Macabre C. (2015). Human papillomavirus-related tumours of the oropharynx display a lower tumour hypoxia signature. Oral Oncol..

[33] Matsumoto T., Bohman S., Dixelius J., Berge T., Dimberg A., Magnusson P., Wang L., Wikner C., Qi J.H., Wernstedt C. (2005). VEGF receptor-2 Y951 signaling and a role for the adapter molecule TSAd in tumor angiogenesis. EMBO J..

[34] Le Buanec H., D’Anna R., Lachgar A., Zagury J.F., Bernard J., Ittele D., d’Alessio P., Hallez S., Giannouli C., Burny A. (1999). HPV-16 E7 but not E6 oncogenic protein triggers both cellular immunosuppression and angiogenic processes. Biomed. Pharmacother..

[35] Chen W., Li F., Mead L., White H., Walker J., Ingram D.A., Roman A. (2007). Human papillomavirus causes an angiogenic switch in keratinocytes which is sufficient to alter endothelial cell behavior. Virology.

[36] Sturgis E.M., Ang K.K. (2011). The epidemic of HPV-associated oropharyngeal cancer is here: Is it time to change our treatment paradigms?. J. Natl. Compr. Cancer Netw..

[37] Vassilakopoulou M., Psyrri A., Argiris A. (2015). Targeting angiogenesis in head and neck cancer. Oral Oncol..

[38] Hsu H.W., Wall N.R., Hsueh C.T., Kim S., Ferris R.L., Chen C.S., Mirshahidi S. (2014). Combination antiangiogenic therapy and radiation in head and neck cancers. Oral Oncol..

[39] Li J., Huang S., Armstrong E.A., Fowler J.F., Harari P.M. (2005). Angiogenesis and radiation response modulation after vascular endothelial growth factor receptor-2 (VEGFR2) blockade. Int. J. Radiat. Oncol. Biol. Phys..

[40] Cerami E., Gao J., Dogrusoz U., Gross B.E., Sumer S.O., Aksoy B.A., Jacobsen A., Byrne C.J., Heuer M.L., Larsson E. (2012). The cBio cancer genomics portal: An open platform for exploring multidimensional cancer genomics data. Cancer Discov..

[41] Gao J., Aksoy B.A., Dogrusoz U., Dresdner G., Gross B., Sumer S.O., Sun Y., Jacobsen A., Sinha R., Larsson E. (2013). Integrative analysis of complex cancer genomics and clinical profiles using the cBioPortal. Sci. Signal..

[42] Farhang Ghahremani M., Goossens S., Haigh J.J. (2013). The p53 family and VEGF regulation: "It’s complicated". Cell Cycle.

[43] Blazquez C., Cook N., Micklem K., Harris A.L., Gatter K.C., Pezzella F. (2006). Phosphorylated KDR can be located in the nucleus of neoplastic cells. Cell Res..

[44] Domingues I., Rino J., Demmers J.A., de Lanerolle P., Santos S.C. (2011). VEGFR2 translocates to the nucleus to regulate its own transcription. PLoS ONE.

[45] Meyer R.D., Srinivasan S., Singh A.J., Mahoney J.E., Gharahassanlou K.R., Rahimi N. (2011). PEST motif serine and tyrosine phosphorylation controls vascular endothelial growth factor receptor 2 stability and downregulation. Mol. Cell Biol..

[46] Spardy N., Covella K., Cha E., Hoskins E.E., Wells S.I., Duensing A., Duensing S. (2009). Human papillomavirus 16 E7 oncoprotein attenuates DNA damage checkpoint control by increasing the proteolytic turnover of claspin. Cancer Res..

[47] Salazar M., Rojo A.I., Velasco D., de Sagarra R.M., Cuadrado A. (2006). Glycogen synthase kinase-3β inhibits the xenobiotic and antioxidant cell response by direct phosphorylation and nuclear exclusion of the transcription factor Nrf2. J. Biol Chem.

[48] Hoxhaj G., Manning B.D. (2020). The PI3K-AKT network at the interface of oncogenic signalling and cancer metabolism. Nat. Rev. Cancer.

[49] Chowdhry S., Zhang Y., McMahon M., Sutherland C., Cuadrado A., Hayes J.D. (2013). Nrf2 is controlled by two distinct beta-TrCP recognition motifs in its Neh6 domain, one of which can be modulated by GSK-3 activity. Oncogene.

[50] Zhao D., Pan C., Sun J., Gilbert C., Drews-Elger K., Azzam D.J., Picon-Ruiz M., Kim M., Ullmer W., El-Ashry D. (2015). VEGF drives cancer-initiating stem cells through VEGFR-2/Stat3 signaling to upregulate Myc and Sox2. Oncogene.

[51] Zhang M., Kumar B., Piao L., Xie X., Schmitt A., Arradaza N., Cippola M., Old M., Agrawal A., Ozer E. (2014). Elevated intrinsic cancer stem cell population in human papillomavirus-associated head and neck squamous cell carcinoma. Cancer.

[52] Reid P., Wilson P., Li Y., Marcu L.G., Staudacher A.H., Brown M.P., Bezak E. (2017). In vitro investigation of head and neck cancer stem cell proportions and their changes following X-ray irradiation as a function of HPV status. PLoS ONE.

[53] Jinesh G.G., Manyam G.C., Mmeje C.O., Baggerly K.A., Kamat A.M. (2017). Surface PD-L1, E-cadherin, CD24, and VEGFR2 as markers of epithelial cancer stem cells associated with rapid tumorigenesis. Sci. Rep..

