# Supplementary material for: Comprehensive Analysis of VEGFR2 Expression in HPV-Positive and -Negative OPSCC Reveals Differing VEGFR2 Expression Patterns
Source: Cancers (Basel). 2021 Oct 18;13(20):5221. doi: 10.3390/cancers13205221 (PMC8533978; doi:10.3390/cancers13205221)
Supplement: Supplementary file 1 [file cancers-13-05221-s001.zip › cancers-1373248-supplementary.pdf]

Supplementary Material

Supplementary Information

Antigen retrieval of the deparaffinized sections was performed by heating at 95°C in 1 mM EDTA buffer (pH 8.0) for 15 min. After treatment with 0.25%-Triton-X 100 detergent solution, the sections were incubated with blocking solution containing 5% normal goat serum (NGS; Vector, Burlingame, USA) for 30 minutes. The sections were incubated with the appropriate first primary antibody (guinea-pig monoclonal anti-human CD31 (1:800; gift from Prof. Dr. M. Koch, Cologne), mouse anti-human polyclonal p16 (1:50; BD Biosciences, Heidelberg, Germany), mouse anti-human polyclonal p53 (1:25; biologo, Kronshagen, Germany) and mouse anti-human ALDH1A1 (1:500; sc-374076, sc-374149 Santa Cruz Biotechnology Inc., California, USA), overnight at 4°C. (Since the established antibody was no longer available, two new clones that showed the same results were evaluated.) Subsequently, the sections were incubated with the corresponding first secondary antibody (biotinylated goat anti-guinea-pig IgG (1:500; Vector), biotinylated goat anti-mouse IgG (p16 1:30, p53 1:20, ALDH1A1 1:300; Vector) for 60 minutes. Then these were incubated with 488-conjugated-NeutrAvidin (1:1000; Thermo Scientific, Massachusetts, USA) for 60 minutes. After treatment with 5% NGS blocking solution, the incubation with the second primary antibody rabbit anti-human VEGFR2 (1:500, ALDH1A1 1:200; 55B11 Cell Signalling Technology, Frankfurt am Main, Germany) was carried out overnight at 4°C. Afterwards, the sections were incubated with the second secondary antibody DyLight-550-conjugated goat anti-rabbit IgG (1:300, ALDH1A1 1:100; Thermo Scientific) for 60 minutes. The sections were incubated with the chromatin marker DRAQ5 (1:2000; Cell Signaling Technology) for 15 min in the dark to identify the cell nuclei and covered with Aqua-Poly/Mount (Polysciences, Hirschberg an der Bergstraße, Germany).

Supplementary figures

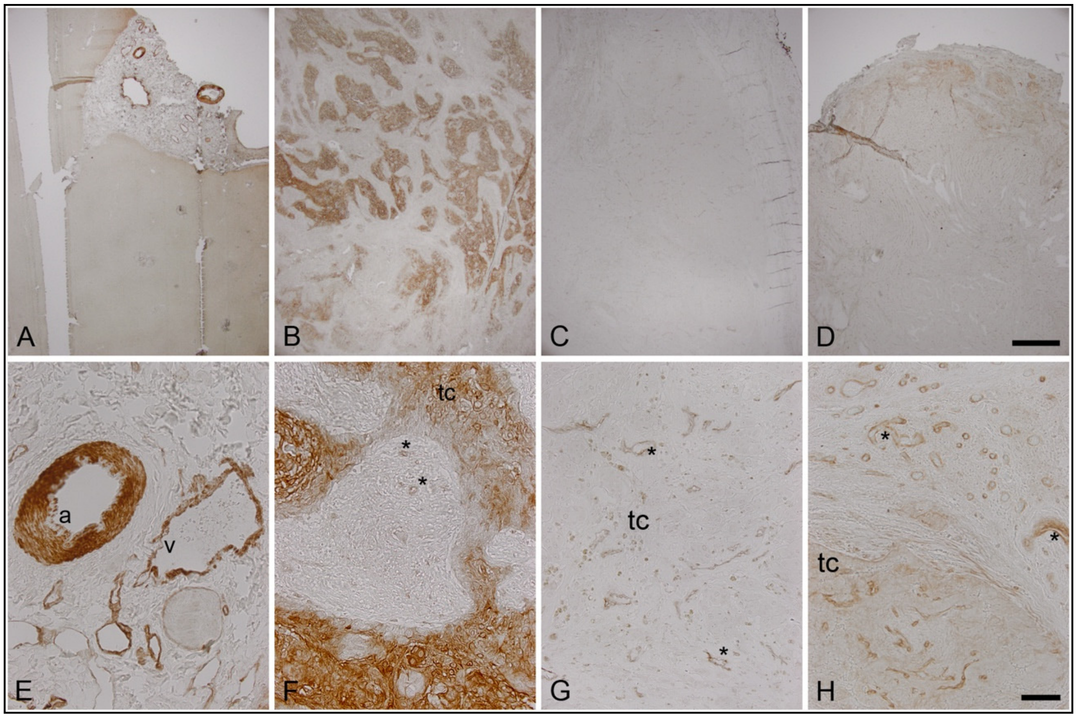

Figure S1. Immunohistochemical positive controls of VEGFR2 expression in tissue samples of liver, cervix squamous cell carcinoma, melanoma, papillary thyroid carcinoma. (**A, E**) Immunohistochemical staining against VEGFR2 of a positive control tissue section of liver to test the localisation of VEGFR2 in blood vessels. (**A**) Overview and (**E**) details. VEGFR2 is detected in arteries (**a**) and veins (**v**). (**B–H**) Immunohistochemical staining against VEGFR2 of positive control tumour tissue sections of cervix squamous carcinoma, melanoma and papillary thyroid carcinoma. (**B–D**) Overview and (**F–H**)

details). VEGFR2 is detected in blood vessels (asterisk) and tumour cells (**tc**) of cervix squamous cell carcinoma (**B, F**), melanoma (**C, G**) and papillary thyroid carcinoma (**D, H**). a = artery, v = vein, tc = tumour cells. Scale bars: A–D 1 mm, E-F 100 μm.

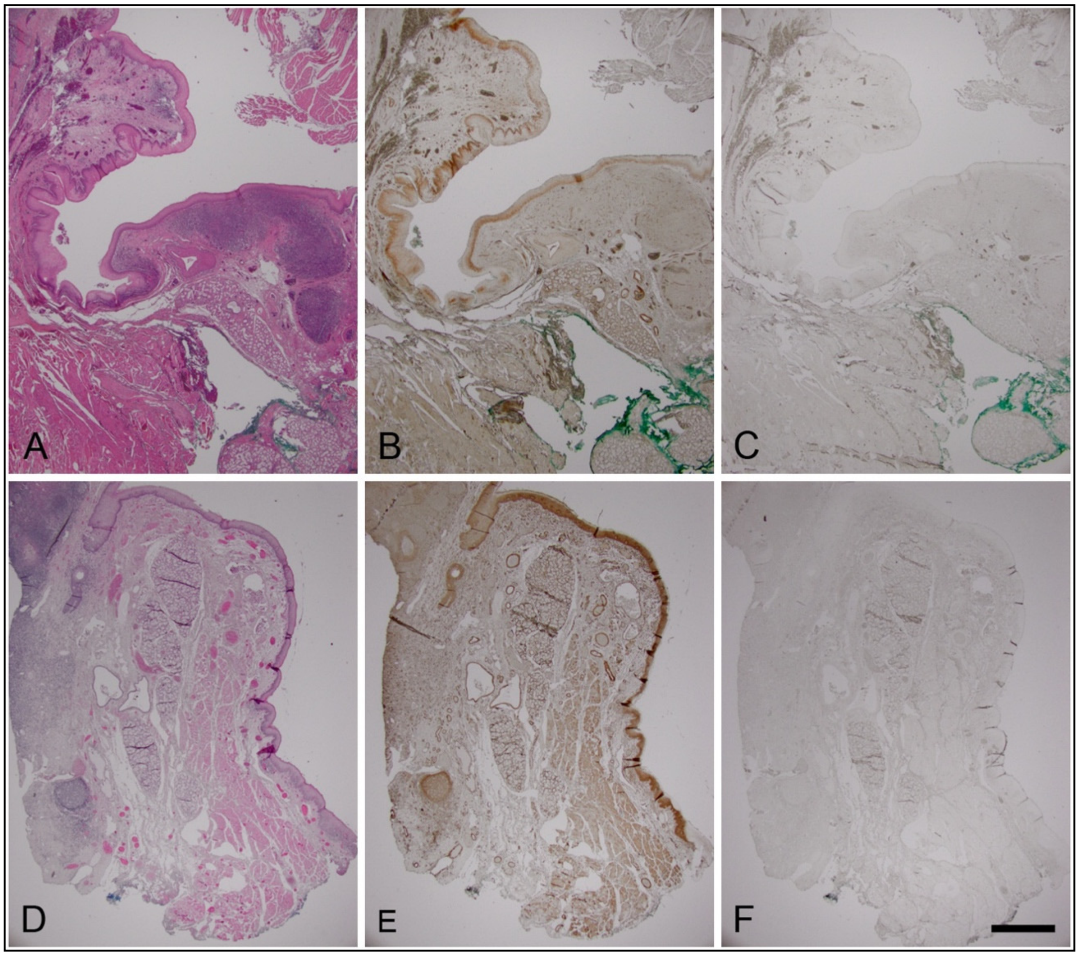

Figure S2. Immunohistochemical controls of the secondary antibodies and the detection system. (**A–C**) Consecutive slides of a representative HPV-positive and (**D–F**) HPV-negative OPSCC. (**A, D**) Histopathological characterisation by H&E staining, (**B, E**) immunohistochemical staining against VEGFR2, (**C, F**) control section incubated without VEGFR2 antibody. In comparison to the VEGFR2-immunoreactive sections (**B, E**), no immunohistochemical localisation is detected in the control section without primary antibody (**C, F**). Scale bar: 1 mm.

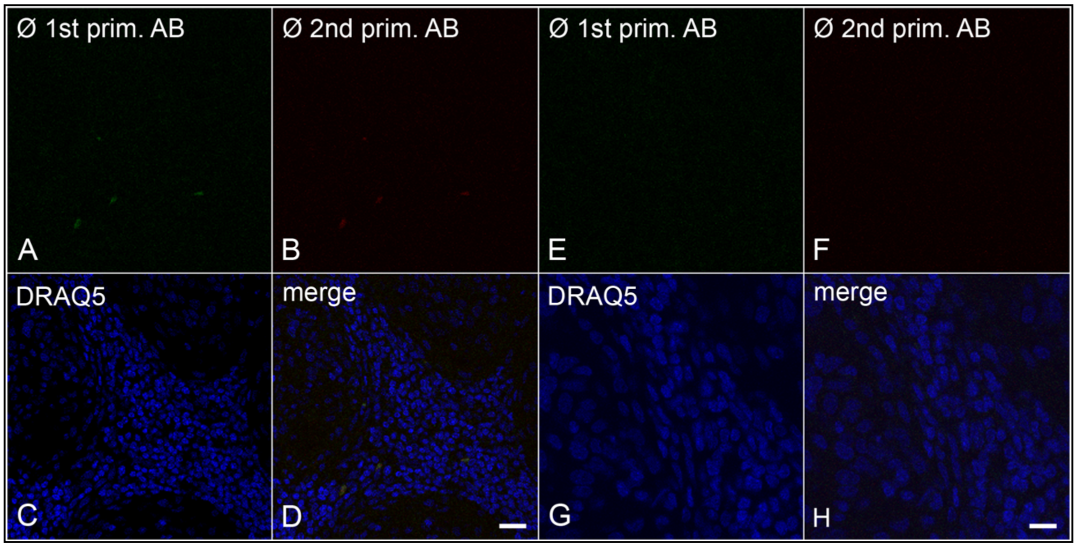

Figure S3. Control of the confocal double immunofluorescence detection system. (**A–D**) Overview and (**E–F**) details. In double immunofluorescence control incubations without first (**A, E**) and second primary antibodies (**B, F**), no specific staining is detectable (**D, H**). Tumour cell nuclei are stained with DRAQ5 (**C, G**). Single erythrocytes within blood vessels show autofluorescence (**D**). AB = antibody, ∅ = without. Scale bars: 20 µm.

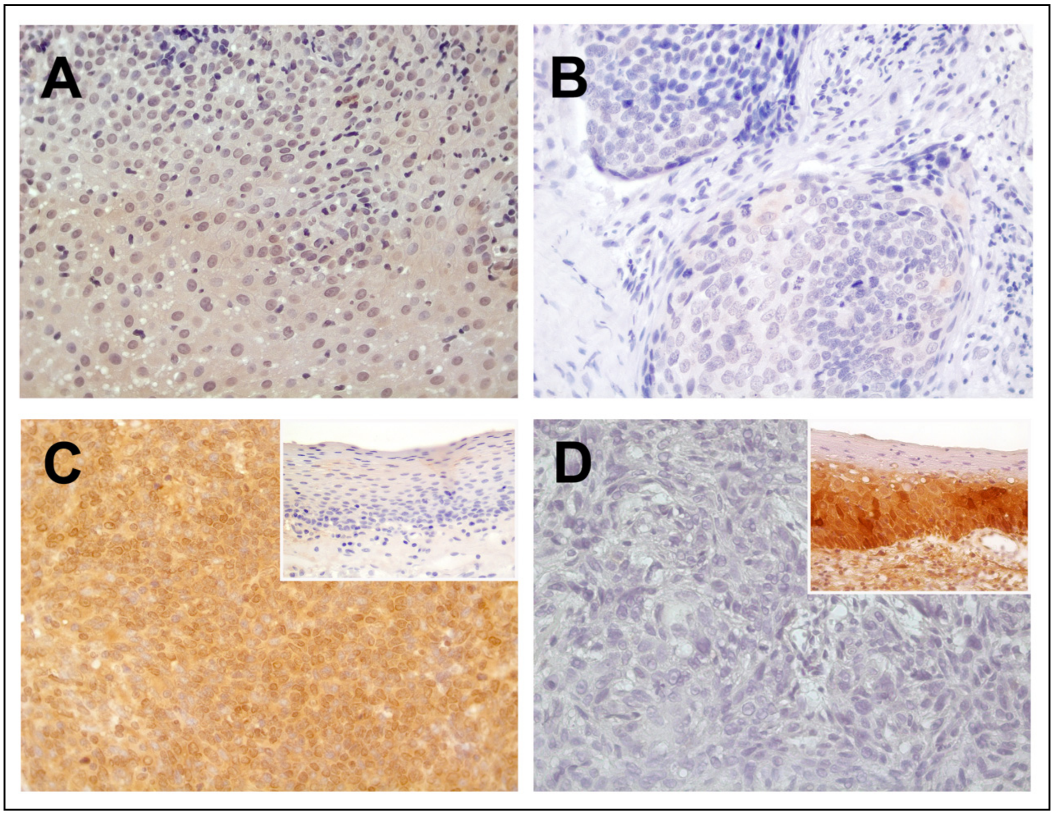

Figure S4. Representative immunohistochemical stainings of NRF2 (**A, B**) and AKR1C3 (**C, D**) in tumour tissue samples. (**A**) Tumour cells with positive nuclear staining against NRF2. (**B**) Lack of NRF2-staining. (**C**) AKR1C3-positive tumour tissue. (**D**) Negative AKR1C3-staining. Insets: adjacent normal squamous epithelium. *V* = x400.

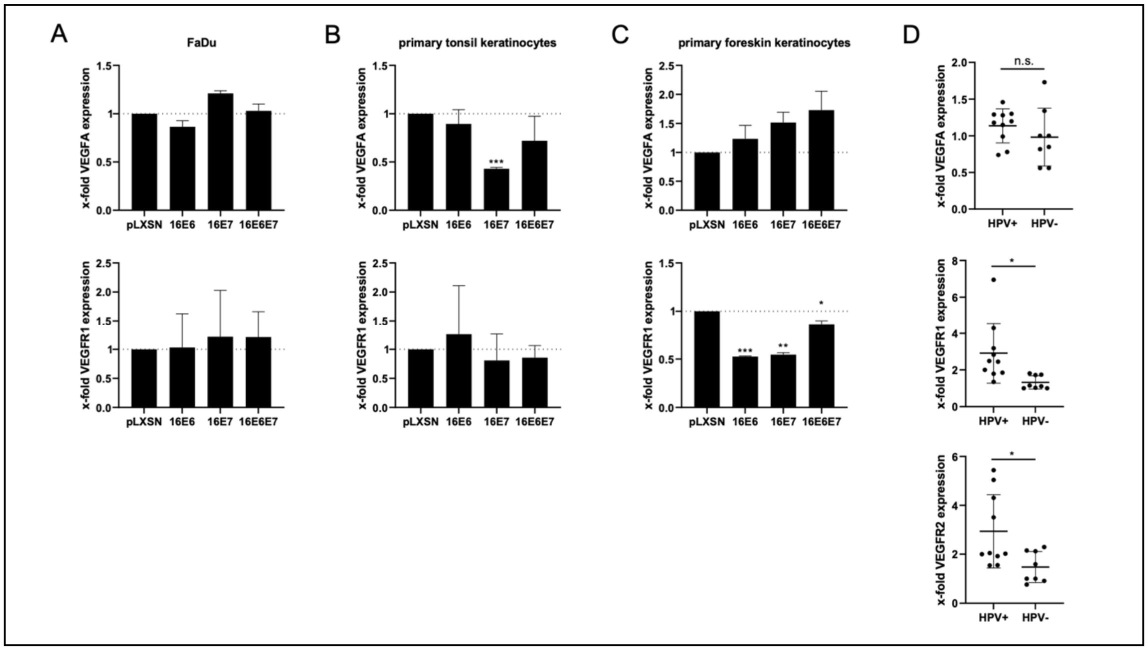

Figure S5. MRNA expression of VEGFA, VEGFR1 and VEGFR2 were measured using reverse transcribed total cellular RNA from (**A**) FaDu cells, (**B**) primary tonsil keratinocytes, (**C**) primary foreskin keratinocytes and (**D**) HPV-negative or -positive OPSCC by RT-qPCR and normalized to HPRT1 mRNA levels (n = 3 independent experiments performed in duplicate). Error bars represent standard deviations. *, $p < 0.01$; **,$p < 0.001$; ***, $p < 0.0001$; n.s., No significant difference.

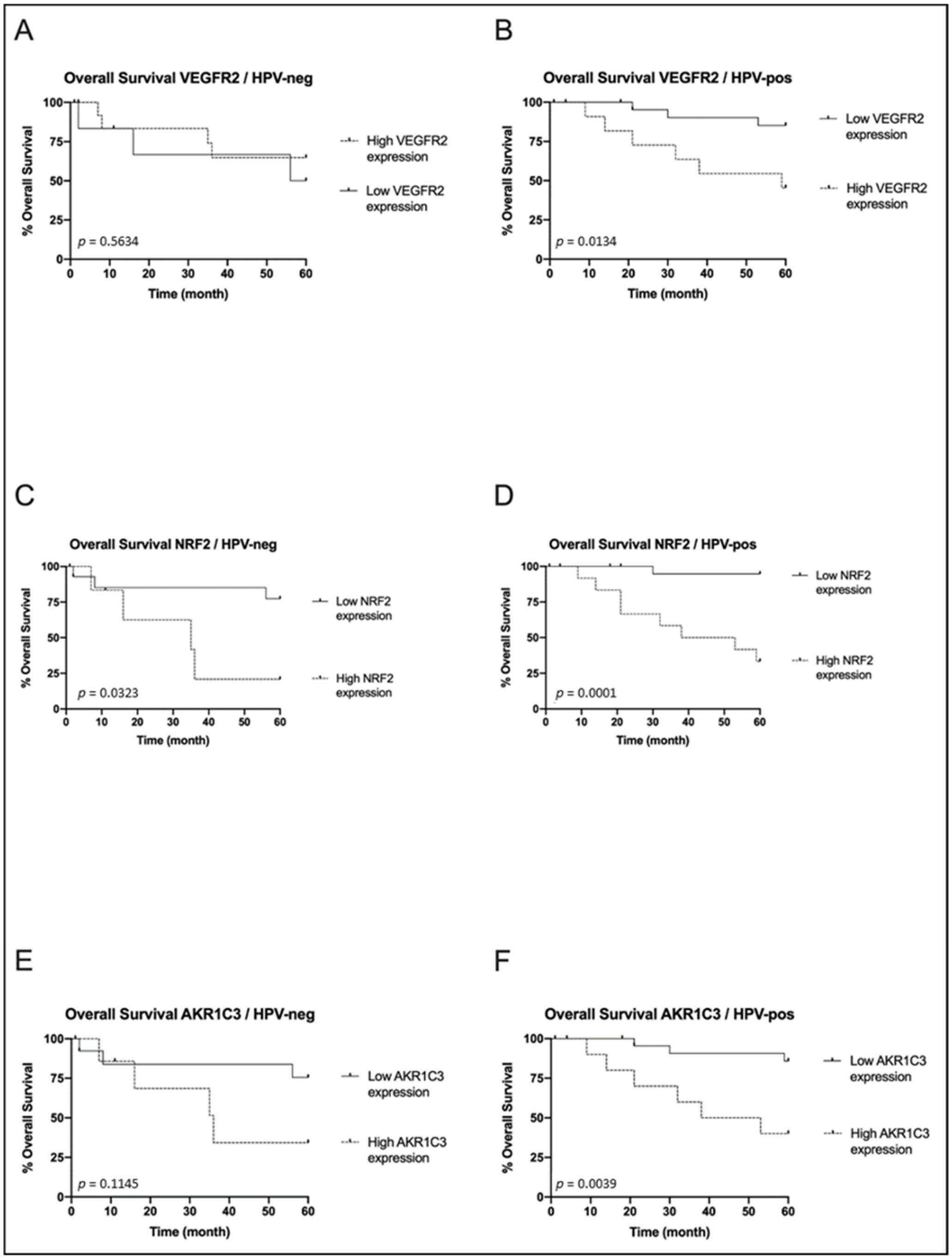

Figure S6. Univariate survival analysis for VEGFR2 (**A, B**), NRF2 (**C, D**) and AKR1C3 (**E, F**) expression-status separated by HPV-status. Kaplan–Meier plots for overall survival (OS) in patients with low vs. high protein expression. p-value was derived by log-rank/Mantel-Cox test.

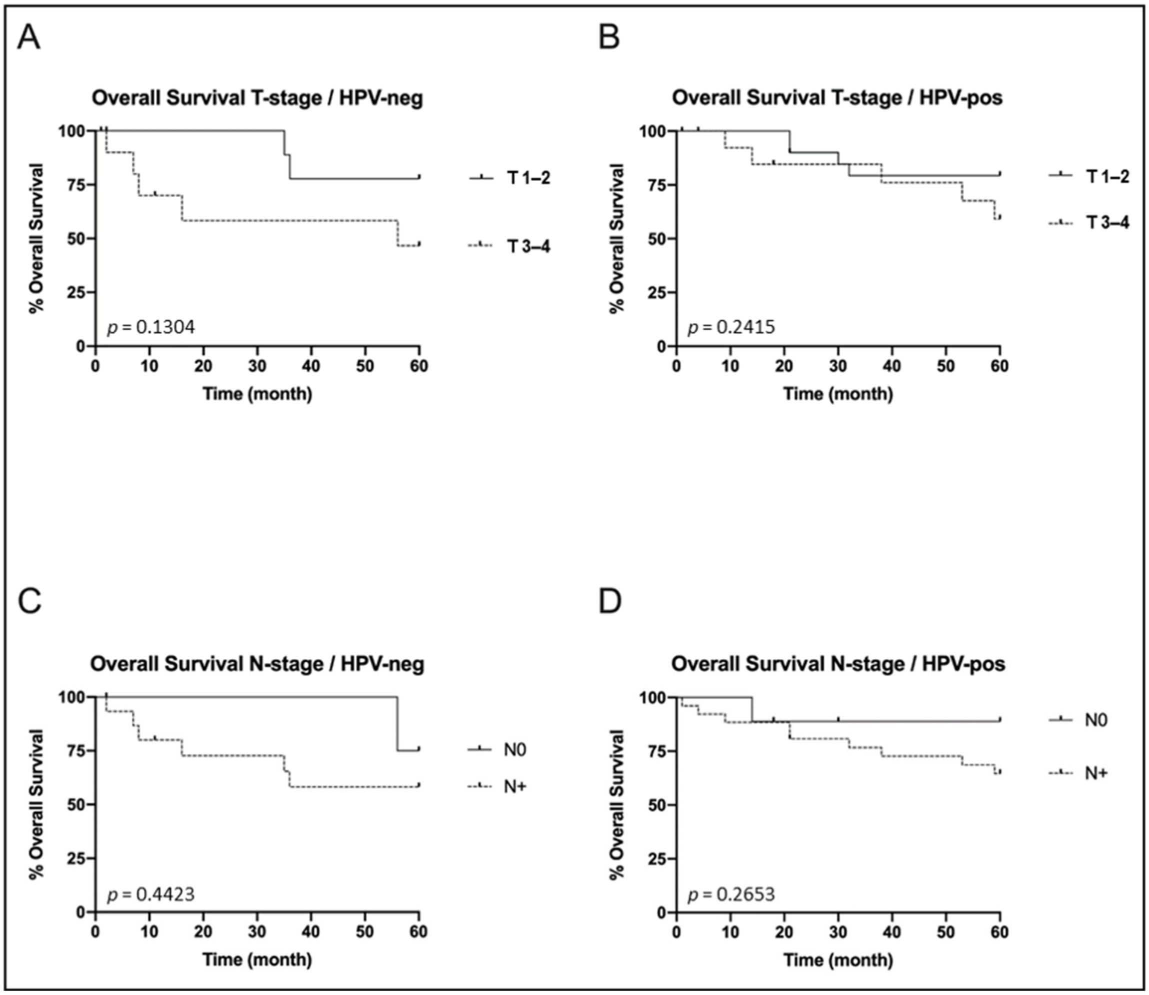

Figure S7. Univariate survival analysis for T-stage (**A, B**) and N-stage (**C, D**) separated by HPV-status. Kaplan–Meier plots for overall survival (OS) in patients with T 1–2 *vs.* T 3–4 and N0 *vs.* N+. *p* value was derived by log-rank/Mantel-Cox test.

Supplementary table

Table S1. Univariate survival analysis.

Samples analysed	Parameters	Group	No.	Overall Survival (OS)		
				Hazard ratio	95% CI	*p*-Value*
**Complete collection**						
	All		55			
	T stage	T1–2	26	0.4	0.1–1.0	**0.049**
		T3–4	29			
	N stage	N0	41	0.5	0.2–1.4	0.176
		N+	14			
	VEGFR2	VEGFR2 low	25	0.4	0.2–1.1	0.091
		VEGFR2 high	30			
	NRF2	NRF2 low	19	0.1	0.1–0.3	**< 0.0001**
		NRF2 high	37			
	AKR1C3	AKR1C3-	18	0.2	0.1–0.5	**0.001**

		AKR1C3+	38			
**HPV-positive**						
	T stage	HPV+/T1–2	20	0.433	0.11–1.7	0.242
		HPV+/T3–4	15			
	N stage	HPV+/N0	9	0.433	0.11–1.9	0.265
		HPV+/N+	26			
	VEGFR2	HPV+/VEGFR2 low	23	0.162	0.04–0.69	**0.013**
		HPV+/VEGFR2 high	12			
	NRF2	HPV+/NRF2 low	23	0.059	0.01–0.2	**< 0.0001**
		HPV+/NRF2 high	12			
	AKR1C3	HPV+/AKR1C3-	25	0.107	0.02–0.5	**0.004**
**HPV-negative**						
	T stage	HPV-/T1–2	10	0.3	0.1–1.4	0.130
		HPV-/T3–4	11			
	N stage	HPV-/N0	5	0.5	0.1–2.8	0.442
		HPV-/N+	15			
	VEGFR2	HPV-/VEGFR2 low	7	1.6	0.3–7.8	0.563
		HPV-/VEGFR2 high	13			
	NRF2	HPV-/NRF2 low	14	0.1	0.1–0.8	**0.032**
		HPV-/NRF2 high				
	AKR1C3	HPV-/AKR1C3-	13	0.3	0.1–1.4	0.115
		HPV-/AKR1C3+	8			

* *p*-Value calculated by log-rank (Mantel-Cox)test. Bold: significant values ≤ 0.050.
